# *Clostridium sordellii* Toxic Shock Syndrome: A Case Report and Review of the Literature

**DOI:** 10.1155/S1064744996000087

**Published:** 1996

**Authors:** Randy C. Sosolik, Beverley A. Savage, Luis Vaccarello

**Affiliations:** 1 Department of Pathology Ohio State University Medical Center 129 Hamilton Hall 1645 Neil Avenue Columbus OH 43210 USA; 2 Department of Obstetrics and Gynecology Ohio State University Medical Center Columbus OH USA

## Abstract

*Background:* Since the 1980s, there have been isolated reports of a toxic shock syndrome associated with *Clostridium sordellii* necrotizing subcutaneous infections during the puerperium. Relatively localized fascial and muscle necrosis is noted at the surgical incision sites. However, circulating toxins produce marked edema, resulting in shock and cardiovascular collapse. Despite aggressive surgical and supportive therapy, all postpartum cases thus far have been fatal.

*Case:* A 24-year-old primipara developed an episiotomy infection which progressed to involve the underlying fascia and muscle. Despite early and adequate debridement of the devitalized tissue, she developed anasarca, marked leukocytosis, refractory hypotension, hypothermia, and a persistent coagulopathy, and expired on postpartum day 5. The cultures from the excised tissue grew *C. sordellii* All blood cultures were negative.

*Conclusion:* Treatment modalities aimed solely at the eradication of the microbe and removal of necrotic tissue, although essential components of therapy, have proved inadequate. Future efforts should be directed toward neutralization or elimination of the circulating exotoxins responsible for the systemic shock.


Infectious Diseases in Obstetrics and Gynecology 4:31–35 (1996)
© 1996 Wiley-Liss, Inc.

### *Clostridium sordellii* Toxic Shock Syndrome: A Case Report and Review of the Literature

Randy C. Sosolik, Beverley A. Savage, and Luis Vaccarello

*Departments of Pathology (R.C.S.) and Obstetrics and Gynecology (B.A.S., L.V.), Ohio State University Medical Center, Columbus, OH*

ABSTRACT

***Background:*** **Since the 1980s, there have been isolated reports of a toxic shock syndrome associated with *Clostridium sordellii* necrotizing subcutaneous infections during the puerperium. Relatively localized fascial and muscle necrosis is noted at the surgical incision sites. However, circulating toxins produce marked edema, resulting in shock and cardiovascular collapse. Despite aggressive surgical and supportive therapy, all postpartum cases thus far have been fatal.**

***Case:*** **A 24-year-old primipara developed an episiotomy infection which progressed to involve the underlying fascia and muscle. Despite early and adequate debridement of the devitalized tissue, she developed anasarca, marked leukocytosis, refractory hypotension, hypothermia, and a persistent coagulopathy, and expired on postpartum day 5. The cultures from the excised tissue grew *C. sordellii*. All blood cultures were negative.**

***Conclusion:*** **Treatment modalities aimed solely at the eradication of the microbe and removal of necrotic tissue, although essential components of therapy, have proved inadequate. Future efforts should be directed toward neutralization or elimination of the circulating exotoxins responsible for the systemic shock.** © 1996 Wiley-Liss, Inc.

KEY WORDS

Anasarca, lethal toxin, myonecrosis, anaerobe

*Clostridium sordellii* is an anaerobic inhabitant of the soil and gastrointestinal tract. Since its discovery in 1922,[1] this species has been occasionally implicated (usually with other histotoxic pathogens) as a cause of gas gangrene. Recently, however, there have been reports of a distinct "toxic shock-like" entity attributed to soft-tissue infections with *C. sordellii* alone.[2-4] The clinicopathologic manifestations and significant morbidity and mortality described with these infections have been linked to the elaboration of 2 unique exoproteins: edema-producing, or lethal, toxin (LT) and hemorrhagic toxin (HT). More specifically, strains with the capacity to produce LT and HT have been associated with puerperal wound infections that are accompanied by anasarca and a clinical picture of septic shock. We report a case of an episiotomy infection with *C. sordellii* and the resultant syndrome as well as a review of the current literature.

### CASE REPORT

A 24-year-old $G_1P_0$ had a post-dates induction at 41-3/7 weeks gestation. The antepartum course was uncomplicated. Labor was induced with Pitocin (Parke-Davis, Morris Plains, NJ) and artificial rupture of the membranes. After a 9.5-h 1st stage of labor, the patient pushed for 3 h. The forceps delivery from +3 station, occiput anterior, was complicated by a 4th-degree midline episiotomy.

Within 24 h postpartum, the patient had increasing perineal pain. Edema was noted around her eyes. On postpartum day 2, her vulva was markedly

Address correspondence/reprint requests to Dr. Randy C. Sosolik, Department of Pathology, Ohio State University Medical Center, 129 Hamilton Hall, 1645 Neil Avenue, Columbus, OH 43210.

Received August 11, 1995

Accepted January 3, 1996

swollen and exquisitely tender to touch. An examination under anesthesia revealed symmetric labial induration that extended back toward the gluteal region. The vagina and uterus were not involved. No abscess, hematoma, or evidence of crepitus was detected. Small vesicles were noted on the perineum. A small amount of purulent material was noted at the inferior end of the episiotomy incision. The patient's hemoglobin level, which was 8.8 g/dl immediately postpartum, had increased to 10.5 g/dl without blood transfusions. Her WBC count had risen to 21,000/μl. The serum creatinine was 0.6 mg/dl, blood urea nitrogen was 10.0 mg/dl, and albumin was 2.1 g/dl (normal: 3.8–5.1 g/dl). Intravenous (IV) ampicillin, gentamicin, and clindamycin were given for a presumed episiotomy infection.

On postpartum day 3, the patient's temperature was 38.7°C and her blood pressure was 96/50, with decreased urine output unresponsive to IV fluids. Her WBC count increased to 60,000/μl, hemoglobin rose to 13 g/dl, and serum creatinine was 1.4 mg/dl. The labia were markedly edematous with nonerythematous induration one-third of the way up the buttocks. The patient complained of severe perineal pain, but she had no pus or necrotic areas on examination. An abdominal and pelvic computed tomographic scan showed ascites but no abscess, hematoma, or air-fluid levels. Over the course of the day, she became progressively edematous and developed clinically detectable ascites. Her WBC count had risen to 84,900/μl, hemoglobin increased to 15.2 g/dl, and serum albumin dropped to 1.8 g/dl. The prothrombin time was 12.6 sec (normal range: 10–13 sec), partial thromboplastin time was 54 sec (normal range: 24–34 sec), fibrinogen was 435 mg/dl (normal: 170–375 mg/dl), and platelet count dropped from 250 K/μl predelivery to 88 K/μl (normal range: 150–400 K/μl). She was given IV immunoglobulin and taken to the operating room for a pelvic examination and possible debridement. A radical vulvectomy, partial vaginectomy, and an abdominal/perineal rectosigmoid resection with endcolostomy were performed. There was vulvar infection with areas of necrosis extending into the vagina and rectum. Intraoperatively, 14 units of packed RBCs, 8 units of fresh frozen plasma, 32 units of platelets, and 11 l of crystalloid were administered. The estimated blood loss was 2,500 cc. A Gram-stained touch prep of the excised tissue showed minimal purulence and mixed bacteria. Postoperatively, the antibiotic coverage was changed to vancomycin, imipenem, and high-dose penicillin. Histologic examination of the resected tissue (after fixation) showed acute necrotizing inflammation of the fascia and skeletal muscle, edema, and hemorrhage, with focal abscess formation and infiltration with several different morphotypes of gram-positive and gram-negative bacteria.

The patient continued to deteriorate despite aggressive care. She became hypothermic, hypotensive, and massively edematous, with a persistent coagulopathy. The vesicles, initially present in the perineum only, enlarged to bullous lesions and spread over the trunk and lower extremities. Massive amounts of fluid, epinephrine, phenylephrine hydrochloride and dopamine were required to maintain her blood pressure. One course of hyperbaric oxygen therapy was performed. The surgical site was packed and reinspected frequently, but there was no evidence of further tissue necrosis. Seizure activity commenced on postpartum day 4. She ultimately suffered cardiac arrest and died on postpartum day 5. *C. sordellii* was the predominant anaerobic organism found in the resected necrotic tissue. *Escherichia coli, Staphylococcus lugdunensis, Lactobacillus spp.*, gamma-hemolytic streptococci, and *Bacteroides ovatus* were also isolated.

At the autopsy, in addition to the anasarca (most striking in the face) and cutaneous transparent bullae distributed over the trunk, lower extremities, and arms, serous effusions were noted in the pleural, peritoneal, and pericardial cavities. The remaining pelvic-floor musculature showed minimal residual focal necrosis and the intraabdominal viscera were grossly and histologically unremarkable. Importantly, there was no evidence of tissue destruction away from the pelvic region. Antemortem and postmortem blood cultures were negative for *C. sordellii* or any other pathogens.

### DISCUSSION

Only 5 similar obstetric cases have been documented in the recent literature.[5–8] The patients described therein shared clinical courses and pathologic manifestations nearly identical to the patient presented here (Table 1). The unique pathogenicity of this clostridial species has evolved over several decades. In 1929, Hall[9] demonstrated that the virulence of *C. sordellii* was due to a toxin (beta toxin) that caused severe, gelatinous edema in animal

**TABLE 1. Commonly reported features of *C. sordellii* toxic shock syndrome**

| |
|---|
| Clinicopathologic findings |
| Previously healthy females with recent "clean" obstetric wounds or incisions |
| Gastrointestinal distress and generalized weakness at presentation |
| Rapidly spreading edema that progresses to anasarca (edema initially localized to perineum or surgical site) |
| Rapid deterioration in cardiovascular status with progressive, refractory hypotension |
| Serous pleural effusions and ascites |
| Lack of fever or hypothermia |
| Varying degrees of coagulopathy |
| Minimal purulent discharge from infection site |
| Absence of other infectious causes for toxic shock syndrome |
| Tissue necrosis localized to area surrounding wound |
| Laboratory findings |
| Leukocytosis with marked left shift |
| Hemoconcentration |
| Abnormal coagulation parameters |
| *C. sordellii* as the principal anaerobic isolate from tissue |
| Anaerobic and aerobic blood cultures negative for pathogens, including *C. sordellii* |

models. Beta toxin was subsequently divided into 2 activities by Arseculeratne et al.[10] in 1969: edema-producing, or lethal, toxin (LT) and hemorrhagic toxin (HT). He[10] noted that the intradermal injection of different preparations of cell-free extracts into guinea pigs induced either lethal edema with moderate hemorrhage (LT) or extensive hemorrhage with minimal or no mortality (HT). Since that time, the 2 toxins responsible for these physiologic derangements have been further characterized. In 1988, Martinez and Wilkins[11] purified HT and proved that it exhibited cross-reactivity with *C. difficile* toxin A (enterotoxin). At a molecular weight of 525 kD, HT demonstrated cytotoxicity in vitro and lethality to mice and induced hemorrhage, mucus, and fluid accumulation in isolated rabbit ileal loops. However, as its name implies, LT appears to be primarily responsible for the morbidity and mortality linked to *C. sordellii* infections. Characterized by Popoff[12] in 1987, LT (250 kD) was lethal for mice by intraperitoneal injection and cytotoxic for Vero cells. It produced erythema and edema when injected intradermally in guinea pigs and caused moderate serous fluid accumulation in guinea pig intestinal loops. LT is antigenically similar to *C. difficile* toxin B,[12] and both alter the phosphorylation pattern of cellular proteins.[13] Antiserum against *C. difficile* toxins A and B has been shown to neutralize the cytotoxicity and lethality of toxin-positive *C. sordellii* culture filtrates.[14]

Although this report adds to the list of postpartum surgical wound infections attributed to toxin-producing strains of this microbe, other rare cases not related to obstetric wounds or incisions further illustrate the pathogenicity of *C. sordellii.* Hogan and Ireland[15] described a case of acute spontaneous endometritis that resulted in cardiovascular collapse and death approximately 18 h after presentation. *C. sordellii* was the sole organism isolated from the endometrial tissue. The necropsy showed mild subcutaneous edema, serous ascites, and bilateral pulmonary edema, but no evidence of infection in the pelvic organs or gastrointestinal tract. Grimwood et al.[4] reported 2 "firsts" with regard to *C. sordellii* soft-tissue infections: 1) infection in a child and 2) survival. A 4-year-old girl presented with a red, swollen great toe which she had injured 5 days earlier after jamming it under a door. The tissue from the necrotic nail bed grew *C. sordellii*, and the swelling that initially involved only the great toe began to move up her leg into the buttocks and abdomen. The great toe was eventually excised, and fasciotomies were performed to the level of the knee; however, the underlying subcutaneous tissues and muscles were viable and not involved. With continued antibiotics, the edema eventually improved. She was discharged 30 days after her admission. The investigators[4] suggested that the child's clinical recovery paralleled the appearance of serum antibodies directed against *C. sordellii* lethal toxin.

The in vivo sources for *C. sordellii* are likely to be the gastrointestinal and reproductive tracts. Although gastrointestinal distress was present in most cases reported, the use of antibiotics or other medications, dietary habits, or bowel motility did not appear to play roles in determining the presence of this pathogen. An overgrowth of toxin-positive *C. difficile* and the resultant colitis related to the above-mentioned factors are well documented.[16] *C. sordellii* has been isolated from a small percentage of patients with documented *C. difficile* colitis, but the significance is unknown.[17] In the cervical flora, clostridial colonization has been shown to decrease during late pregnancy and the postpartum period;[18] however, in at least 1 case mentioned earlier (Hogan and Ireland[15]), the genital tract was the most obvious source for the organism. The common features seen in these postpartum patients offer some insight into the pathophysiology of this syndrome. After damage to the mucosa, skin, and other natural barriers during delivery, as in a cesarean delivery or episiotomy, an anaerobic microenvironment may be created because of a decrease in the local blood flow to the damaged tissue and the proliferation of indigenous aerobic flora. If a toxin-secreting strain of *C. sordellii* contaminates the wound and proliferates, tissue necrosis and the production of LT and HT commence. Rapid dissemination of the toxins may occur in view of the overall increased cardiac output directed toward the reproductive organs after birth. As mentioned earlier, this microbe has been recovered from tissue samples but not blood cultures in these patients, which reinforces and further demonstrates that the spread of exotoxins (primarily LT) is the primary instigator of the morbidity and mortality seen with this toxic shock syndrome.

The histotoxic clostridia can produce an array of soft-tissue infections ranging from cellulitis to necrotizing fascitis to frank myonecrosis. Often, the clinicopathologic presentation suggests the identity of the clostridial species, e.g., *C. perfringens* and gas gangrene. The histotoxic exoproteins manufactured by *C. sordellii* result in localized fascial necrosis and myonecrosis which can mimic gas gangrene. An early diagnosis of this or any necrotizing subcutaneous infection based on clinical grounds and an external examination alone can be exceedingly difficult. Consequently, prompt surgical intervention is necessary to 1) determine the extent and nature of the soft-tissue damage, 2) remove the source of toxin production and necrotic tissue, and 3) obtain tissue for histopathologic examination and culture confirmation. Intraoperative frozen-section biopsies have been used to diagnose necrotizing subcutaneous infections early in their course and subsequently decrease mortality, but this technique was not utilized in the cases mentioned here.[19] This technique could prove useful for establishing the presence of myonecrosis associated with *C. sordellii* and the absence of muscle involvement in necrotizing fascitis. In addition, if Gram's stains on involved fresh tissue identify a single morphotype of pathogen, such as staphylococci or streptococci, specific antimicrobial therapy can be initiated. The most common form of necrotizing fascitis, although a separate entity, is microscopically similar to *C. sordellii* infection in that both processes are typified by a paucity of neutrophils and a polymicrobial infiltrate by Gram's stain.[20] Thus, although diagnosing this rare infection before the culture results are known is unlikely, some clinical clues should put this clostridial species in the differential diagnosis. In the case reported here, the gynecologic surgeons considered *C. sordellii* prior to an identification by culture primarily because of the systemic toxicity and profound, rapidly progressive, widespread edema. Leukocytosis, hemoconcentration, and lack of a predominant organism on tissue Gram's stain in the presence of myonecrosis were less specific supporting evidence. *C. sordellii* as a potential etiology was also reinforced through personal communication with an author (James A. McGregor) of 3 previously documented obstetric cases.[5] The possibility of *C. sordellii* infection was communicated to the surgical pathologist who, in turn, prompted the microbiology laboratory to work up all anaerobic isolates, particularly any clostridial species. This latter point should be emphasized because not all laboratories have the capacity to fully speciate some anaerobes and, in some previous reports of necrotizing obstetric wound infections, some clostridial species other than *C. perfringens* were considered contaminants or nonpathogenic organisms.[6,7]

The management of puerperal infections with this clostridial species has been similar to the treatment of *C. perfringens* myonecrosis: surgical debridement of necrotic tissue, broad-spectrum antimicrobials because of the polymicrobic nature of perineal infections, high-dose penicillin, hyperbaric oxygen therapy, and intensive supportive care. Despite this aggressive therapy, all maternal infections thus far have been fatal, and cardiovascular collapse secondary to marked "third space" sequestration of fluid has been the cause of death in all instances.

### CONCLUSIONS

Fortunately, necrotizing subcutaneous infections during the puerperium are rare. In few cases, a prompt diagnosis is often hampered by deceptive clinical manifestations which may delay surgical intervention. Recently, there have been at least 5 documented cases of necrotizing subcutaneous and muscle infections caused by *C. sordellii* in obstetric patients following vaginal or cesarean deliveries. Clostridial soft-tissue infections are typically characterized by widespread undermining of the subcutaneous tissue, myonecrosis, and rapid progression of tissue destruction away from a contaminated wound site. In contrast, puerperal infections with *C. sordellii* are associated with relatively "clean" incisions; they produce localized tissue damage but a profound exotoxin-mediated systemic response characterized by anasarca, refractory hypotension, and marked leukocytosis. The exotoxins (primarily LT) appear to disrupt the vascular integrity, producing extensive "third spacing" and, ultimately, cardiovascular collapse. It appears that debridement and antibiotic therapy do not reverse the chain of events set in motion by the elaboration of toxins in this disease process. Preventative methods such as screening for toxin-positive strains would not be cost-effective in light of the rarity of this complication. Information regarding the pathophysiologic effects of these toxins at the cellular, and even tissue, level is in its infancy. Neutralization or elimination of the exotoxins appears essential because, in one instance, survival was linked to the eventual endogenous production of toxin-neutralizing antibodies.[4] Unfortunately, exogenous antisera are not readily available and its use has not been reported. Another possible therapy could be plasma exchange to decrease the toxin levels; however, the concomitant hypotension may preclude this mode of intervention. The rapid lethality of this rare infectious complication and the minimal data currently available regarding the pathophysiology of the microbe and its exoproteins present a challenge in establishing effective therapeutic options.

### REFERENCES

1. Sordelli A: Un germen anaerobico de las gangrenas gasosas. Rev Assoc Med Argent 35:217–221, 1922.
2. Browdie DA, Davis JH, Koplewitz MJ, Corday L, Leadbetter AW: *Clostridium sordellii* infection. J Trauma 15:515–519, 1975.
3. Hatheway CL: Toxigenic clostridia. Clin Microbiol Rev 1(3):66–98, 1990.
4. Grimwood K, Evans GA, Govender ST, Woods DE: *Clostridium sordellii* infection and toxin neutralization. Pediatr Infect Dis J 9:582–588, 1990.
5. McGregor JA, Soper DE, Lovell G, Todd JK: Maternal deaths associated with *Clostridium sordellii* infection. Am J Obstet Gynecol 161:987–995, 1989.
6. Golde S, Ledger WJ: Necrotizing fascitis in postpartum patients: A report of four cases. Obstet Gynecol 50:670–673, 1977.
7. Ewing TL, Smale LE, Elliott FA: Maternal deaths associated with postpartum vulvar edema. Am J Obstet Gynecol 134:173–179, 1979.
8. Soper DE: Clostridial myonecrosis arising from an episiotomy. Obstet Gynecol 68:26S–28S, 1986.
9. Hall IC: The occurrence of *Bacillus sordellii* in icterohemoglobinuria of cattle in Nevada. J Infect Dis 45:156–162, 1929.
10. Arseculeratne SN, Panabokke RG, Wijesunder S: The toxins responsible for the lesions of *Clostridium sordellii* gas gangrene. J Med Microbiol 2:37–53, 1969.
11. Martinez RD, Wilkins TD: Purification and characterization of *Clostridium sordellii* hemorrhagic toxin and cross-reactivity with *Clostridium difficile* toxin A (enterotoxin). Infect Immun 56:1215–1221, 1988.
12. Popoff MR: Purification and characterization of *Clostridium sordellii* lethal toxin and cross-reactivity with *Clostridium difficile* cytotoxin. Infect Immun 55:35–43, 1987.
13. Ciesielski-Treska J, Ulrich G, Baldacini O, Monteil H, Aunis D: Phosphorylation of cellular proteins in response to treatment with *Clostridium difficile* toxin B and *Clostridium sordellii* toxin L. Eur J Cell Biol 56:68–78, 1991.
14. Martinez RD, Wilkins TD: Comparison of *Clostridium sordellii* toxins HT nd LT with toxins A and B of *C. difficile.* J Med Microbiol 36:30–36, 1992.
15. Hogan SF, Ireland K: Fatal acute spontaneous endometritis resulting from *Clostridium sordellii.* Am J Clin Pathol 91:104–106, 1989.
16. Pothoulakis C, LaMont JT: *Clostridium difficile* colitis and diarrhea. Gastroenterol Clin North Am 22(3):623–637, 1993.
17. Dhamabutra N, Vichivanives P: *Clostridium sordellii* in diarrhoeal stools, its medical significance. J Med Assoc Thailand 74(5):264–270, 1991.
18. Goplerud CP, Ohm MJ, Galask RP: Aerobic and anaerobic flora of the cervix during pregnancy and the puerperium. Am J Obstet Gynecol 126:858–868, 1976.
19. Stamenkovic I, Lew PD: Early recognition of potentially fatal necrotizing fascitis. N Engl J Med 310:1689–1693, 1989.
20. Giuliano A, Lewis F, Hadley K, Blaisdell FW: Bacteriology of necrotizing fascitis. Am J Surg 134:52–57, 1977.
